# Smoking in Patients With Chronic Cardiovascular Disease During COVID-19 Lockdown

**DOI:** 10.3389/fcvm.2022.845439

**Published:** 2022-04-26

**Authors:** Frédéric Chagué, Mathieu Boulin, Jean-Christophe Eicher, Florence Bichat, Maïlis Saint-Jalmes, Amélie Cransac, Agnès Soudry, Nicolas Danchin, Gabriel Laurent, Yves Cottin, Marianne Zeller

**Affiliations:** ^1^Service de Cardiologie, Centre Hospitalier Universitaire, Dijon, France; ^2^Réseau Français d'Excellence de Recherche sur le tabac, la nicotine et les produits connexes, Paris, France; ^3^Département de Pharmacie, Centre Hospitalier Universitaire, Dijon, France; ^4^Département de Recherche Clinique, Centre Hospitalier Universitaire, Dijon, France; ^5^Service de Cardiologie, Hôpital Européen Georges Pompidou, Paris, France; ^6^PEC2, EA 7460, Université Bourgogne Franche-Comté, Dijon, France

**Keywords:** smoking, COVID-19, lockdown, chronic coronary syndrome, congestive heart failure (CHF)

## Abstract

**Objectives:**

This cross-sectional study aims to investigate health-related behaviors including tobacco consumption among patients with cardiovascular diseases (CVD), during the first COVID-19-related lockdown.

**Methods:**

After 5 weeks of COVID-19 lockdown, 220 patients with chronic coronary syndromes (CCS) and 124 with congestive heart failure (CHF) answered a phone questionnaire.

**Results:**

Among these 344 patients, 43 (12.5%) were current smokers, and none had quit during the lockdown. When compared with non-smokers, smokers were 15 years younger, more often diabetic, more likely to live in an urban than a rural lockdown location, and more often in the CCS cohort (*p* = 0.011). Smokers described greater psychological impairment, but their rates of decrease in physical activity and of increase in screen time were similar to non-smokers. More than one-third (13/43) increased their tobacco consumption, which was mainly related to stress or boredom, but not driven by media messages on a protective effect of nicotine.

**Conclusions:**

During the first COVID-19 lockdown, we found a decrease in favorable lifestyle behaviors among patients with CVD. Strikingly, one-third of smokers with CCS or CHF increased their tobacco consumption. Given the major impact of persistent smoking in patients with CVD, this highlights the need for targeted prevention strategies, in particular during such periods.

## Introduction

Cardiovascular disease (CVD), including congestive heart failure (CHF) and chronic coronary syndrome (CCS), and smoking are among the factors that can dramatically worsen prognosis in patients hospitalized for COVID-19 (1). Tobacco smoking is a major reversible risk factor for CVD, and cessation is a major target for prevention. Unfortunately, patients often do not quit smoking after an acute CVD event (2, 3). Although considered as less harmful than smoking, the cardiovascular impact of vaping is still debated (1).

Since the start of the current pandemic, the fear of severe acute respiratory syndrome coronavirus-2 (SARS-CoV2) infection and the strict lockdowns may have generated anxiety and stress, delayed access to care, and favored unhealthy behaviors, such as smoking increase, start or relapse. All of these factors can worsen a CVD patient's long-term prognosis (1, 3–5). On the other hand, the pandemic-related lockdowns were particular situations that may also have potentially favored smoking cessation through fear of illness, the lifting of social barriers, and enabling patients to focus on the health benefits of a healthier lifestyle (6, 7). At the same time, some media outlets spread the unconfirmed information that nicotine could confer a protective effect against COVID-19, thus potentially encouraging patients to smoke (6, 8). While the subject of smoking during the COVID-19 lockdowns has been addressed, investigations in CVD patients are paradoxically very scarce. We hypothesized that smoking rate and related health behaviors could have been modified in patients with CVD during the 2020 lockdown.

## Methods

CLEO-CD (COVID-19 Lockdown Effect On Chronic Diseases) is a cross-sectional study including more than 1200 outpatients with chronic disease from our university hospital in Dijon, France. Among them, 250 CCS subjects were randomly selected from the RICO (observatoire des Infarctus du myocarde de Côte d'Or) survey, which prospectively includes all patients hospitalized for acute myocardial infarction (AMI) in the coronary care unit of our hospital, as previously described (9). Only patients hospitalized for AMI in 2018 and 2019 were selected for inclusion. In addition, 150 CHF outpatients were randomly selected from the Heart Failure Clinic (10). This questionnaire was previously tested on 10 subjects (members of our research unit) as an internal procedure in order to assess compliance (understanding, coherence, reliability), leading to changes in the questions regarding medications and tobacco consumption. Then the questionnaire was tested by phone on eight CCS outpatients and eight CHF outpatients, all non-included in the randomly-selected patients and no changes were found to be necessary. A translated version of the questionnaire addressing tobacco consumption is available in Supplementary File 1 - Questionnaire. A smoker and a vaper were defined as a current tobacco smoker or electronic cigarette user (daily or occasional) at the time of the interview, and an ex-smoker and ex-vaper as having quit any time before the interview. Psychological distress was assessed by the Kessler 6 (K6) score (11). Residence during the lockdown was defined as rural when patients were living in areas with <2,000 inhabitants, and urban when in areas with 2,000 inhabitants or more, in agreement with French demographical definition (https://www.insee.fr/fr/metadonnees/definition/c1501) and as previously described (9). Informed consent was obtained from all of individual participants included in the study.

Because of the nature of the survey, patients were invited to participate and had to give their oral consent before the beginning of the interview.

The present study complied with the Declaration of Helsinki and was approved by the Ethics Committee of the Dijon University Hospital (NCT04390126).

### Statistical Analysis

Continuous variables were expressed as medians and interquartile ranges (IQR) and dichotomous variables as *n* (%). Student *t*-tests or Mann-Whitney tests were used to compare continuous variables, and Pearson's Chi^2^ or Fisher's tests to compare dichotomous data, as appropriate. Current smokers were compared with non-smokers.

## Results

Among the 400 selected patients, 56 declined the interview or were lost to follow-up and 344 questionnaires were finally analyzed, including 220 CCS and 124 CHF; patients with CCS were 3 years younger than those with CHF (*p* = 0.01). The rate of smoking was high (*n* = 43, 12.5%), and smokers were 15 years younger than non-smokers (*p* < 0.001). Population characteristics are summarized in Tables 1, 2. Prevalence of smoking was higher in the CCS than in the CHF group (*p* = 0.011). Smokers were more frequently diabetic, single or divorced, and unemployed than non-smokers, and they were more often screened for COVID-19. In addition, smokers' place of residence during the lockdown tended to be more often urban and they were more likely to be living alone. Feeling and lifestyle behavior of patients according to their smoking status is summarized in Table 3. Among psychological factors, smokers were three times more likely to feel cramped and the psychological distress level (K6 ≥ 8) tended to be higher. Among the 43 current smokers, a high rate [*n* = 10 (30%)] increased their tobacco consumption during the lockdown period. Moreover, during this period, one started to smoke and two had relapsed. Only six patients were vapers, and none was a dual user. Among the ex-smokers, none had quitted since the beginning of the lockdown.

**Table 1:** Population characteristics according to cardiovascular disease.

		**CCS**	**CHF**	***P*-value**
**Population**		**220 (64.0)**	**124 (36.0)**	
Female		66 (29.6)	49 (39.5)	0.08
Age (y)		67 (58–75)	70 (64–82)	0.01
Diagnosis ≥6 months		215 (97.7)	120 (96.8)	0.73
**CCS**				
History of revascularisation		184 (83.6)		
Medications	Antiplatelets agents	200 (91.7)		
	Betablockers	188 (87.0)		
	ACEI or ARB	181 (84.5)		
	Statins	188 (87.0)		
**CHF**				
Type of CHF	HFrEF		87 (70.2)	
	HFmrEF		12 (9.7)	
	HFpEF		25 (20.2)	
Etiology	DCM		50 (40.3)	
	Ischemic		23 (18.5)	
	Others		51 (41.2)	
NYHA Class	I		39 (31.5)	
	II		48 (38.7)	
	III		28 (22.6)	
	IV		9 (7 .3)	

*CCS, chronic coronary syndrome; ACEI, angiotensin converting enzyme inhibitor; ARB, angiotensin receptor blocker; CHF, congestive heart failure; IQR, interquartile range; HFrEF, heart failure with reduced ejection fraction; HFmrEF, heart failure with mildly-reduced ejection fraction; HFpEF, heart failure with preserved ejection fraction; DCM, dilated cardiomyopathy; NYHA, New York Heart Association*.

*n (%) or median (IQR)*.

**Table 2:** Patient characteristics according to smoking status.

	**Total**	**Non-smoker**	**Smoker**	*P*-value^*^
	***N* = 344**	***N* = 301**	***N* = 43**	
**Risk factors**				
Age, years	70 (59–78)	71 (62–79)	56 (52–65)	<0.001
Men	229 (66.6)	197 (65.4)	32 (74.4)	0.243
Diabetes	81 (23.7) {342}	68 (22.7) {299}	13 (30.2)	0.028
BMI, kg/m^2^	27 (24-30) {319}	27 (24-30) {279}	28 (25-31) {40}	0.202
BMI ≥ 25 kg/m^2^	231 (72.4) {319}	200 (71.7) {279}	31 (77.5) {40}	0.442
BMI ≥ 30 kg/m^2^	86 (24.5) {319}	73 (26.2)	13 (32.5)	0.398
Type of CVD	{344}	{301}	{43}	0.011
CCS	220 (64.0)	185 (61.5)	35 (81.4)	
CHF	124 (36.0)	116 (38.5)	8 (18.6)	
History of depression	54 (16.0) {338}	46 (15.5) {297}	8 (19.5) {41}	0.510
COVID-19 screening (RT-PCR)	11 (3.2) {342}	7 (2.3) {299}	4 (9.3)	**0.037**
**Socio-economic status**				
Marital status	{336}	{296}	{40}	0.004
Single	35 (10.4)	26 (8.8)	9 (22.5)	
Divorced	28 (8.3)	22 (7.4)	6 (15.0)	
Married	225 (67.0)	203 (68.6)	22 (55)	
Widower	48 (14.3)	45 (15.2)	3 (7.5)	
Professional activity	{341}	{299}	{42}	<0.001
Current	69 (20.2)	55 (18.4)	14 (33.3)	
Retired	238 (69.8)	222 (74.2)	16 (38.1)	
Unemployed	13 (3.8)	7 (2.3)	6 (14.3)	
Other	21 (6.2)	15 (5.0)	6 (14.3)	
Education	{342}	{300}	{42}	0.112
≥High school diploma	106 (32.1)	97 (33.7)	9 (21.4)	0.133
**Lockdown place**				
Residence area				0.066
Urban	163 (45.3)	137 (45.5)	26 (60.5)	
Rural	181 (54.7)	164 (54.5)	17 (39.5)	
Type of accommodation				0.086
Flat without terrace/garden	47 (12.4)	37 (12.4)	10 (23.3)	
Flat with terrace/garden	66 (18.8)	56 (18.8)	10 (23.3)	
House with garden	228 (68.8)	205 (68.8)	23 (63.5)	
Alone in accommodation	83 (24.4) {340}	68 (22.9) {297}	15 (34.9)	0.087
Number of cohabitants	{340}	{297}	{43}	
Median (IQR)	1 (1–2)	1 (1–2)	1 (0–2)	0.864
Minimum/maximum	0/6	0/6	0/5	

*IQR, interquartile range; BMI, Body Mass Index; CVD, cardiovascular disease; CCS, chronic coronary syndrome; CHF, congestive heart failure; RT-PCR, nasal Reverse Transcriptase-Polymerase Chain Reaction detection for SARS-CoV-2*.

* *p value comparison between smokers and non-smokers*.

*n (%) or median (IQR), {Number of answers}*.

**Table 3:** Patients feeling and behavior according to smoking status during lockdown.

	**Total**	**Non-smoker**	**Smoker**	*P*-value^*^
	***N* = 344**	***N* = 301**	***N* = 43**	
**Psychological factors**				
Lockdown rules compliance	335 [97.7) {343}	294 [98.0) {300}	41 [95.3)	0.264
Feeling cramped	19 [5.6) {337}	13 [4.4) {294}	6 [14)	0.023
Sleep quality/duration change	83 [24.3) {342}	68 [22.7) {300}	15 [35.7) {42}	0.158
Currently feeling:	{342}	{300}	{42}	0.427
Bad	21 [6.1)	16 [5.3)	5 [11.9)	
Fairly good	75 (21.9)	67 (22.3)	8 (19.0)	
Well	175 (51.2)	154 (51.3)	21 (50.0)	
Very well	71 (20.8)	63 (21.0)	8 (19)	
Feeling less well (compared to before lockdown)	75 (21.9) {342}	65 (21.7) {300}	10 (32) {42}	0.743
Kessler score	2 (0–4) {337}	2 (0–4) {294}	2 (0–4)	0.633
K6 ≥ 8	37 (11.0)	29 (9.9)	8 (18.6)	0.079
**Health behavior change**				
Physical activity	{341}	{298}		0.466
Same	171 (50.1)	153 (51.3)	18 (41.9)	
Decreased	147 (43.1)	125 (41.9)	22 (51.2)	
Increased	23 (6.7)	20 (6.7)	3 (7.0)	
Alcohol intake	284	245	39	0.341
Same	242 (85.2)	210 (85.7)	32 (82.1)	
Decreased	27 (9.5)	24 (9.8)	3 (7.7)	
Increased	15 (6.7)	11 (4.5)	4 (10.3)	
Screen time increase	155 (45.3) {342}	131 (43.7) {300}	24 (57.1) {42}	0.10
Weight	{343}	{301}	{42}	0.01
Same	223 (65.0)	204 (67.8)	19 (45.2)	
Decreased	43 (12.5)	33 (11.0)	10 (23.8)	
Increased	77 (22.4)	64 (21.3)	13 (31)	
**Tobacco consumption**				
Same			21 (48.8)	
Decreased			9 (20.9)	
Increased (or started)			13 (30.2)	
Cause of increase/start smoking			{12}	
Stress	–	–	7 (58.3)	
Boredom	–	–	3 (25.0)	
Other			2 (16.7)	
**Electronic cigarette**	6 (1.8) {333}	6 (2.1) {290}	0 (0.0)	1
With nicotine	1 (20) {5}	1 (20) {5}	0	

*IQR, interquartile range*.

* *p-value comparison between smokers and non-smokers*.

*n (%) or median (IQR), {Number of answers}*.

Stress was the most commonly cited cause of smoking, followed by boredom. Lifestyle changes, including physical activity, alcohol consumption, and increase in screen time were similar for the two groups. In contrast, smokers had a much higher rate of weight variations, either for increase or for decrease, when compared with non-smokers. At the time of the interview, 29 patients reported the use of telemedicine, 16 in the CCS group (7.3%) and 13 in CHF group (10.5%); the difference was non-significant (p = 0.317). As tobacco quitting may have been encouraged during these sessions, we assume that such advice may have been given in the same way in both groups.

Among the 344 patients, three patients developed conditions highly suggesting a COVID-19 (anosmia and/or ageusia associated with fever and cough) and underwent PCR testing (unknown timing according to the symptoms), of whom only one was positive. Eight other patients underwent PCR testing, of whom all were negative. Among them, four had no symptoms, neither contact with any COVID-19 patient.

Among the patients with CCS, 13 declared an increase of symptoms of angina, of whom two were smokers. One of them did not report a change in smoking behavior, the other declared a reduction in tobacco consumption.

The subgroup analysis among smokers showed that the decrease in physical activity and the increase in screen time were more common in urban than in rural areas (61.5 vs. 35.3%, *p* = 0.092 and 69.2 vs. 37.5%, *p* = 0.044, respectively). Although not significant, tobacco consumption increased less frequently among rural vs. urban patients (17.6 and 38.5%, *p* = 0.187).

## Discussion

Smoking cessation is associated with major health benefits and some studies even suggest a favorable effect on biological age (12). Although smoking cessation is one of the key targets for secondary prevention in CVD, we found a high rate of current smokers (12.5%) among French CVD patients interviewed during the first lockdown (March-May 2020), consistent with smoking prevalence in CAD patients from contemporary European surveys (2, 13).

Relations between tobacco smoking and COVID-19 are controversial. Comorbidities including tobacco-induced diseases are associated with severe forms of COVID-19 and smokers are at higher risk of poor outcomes when infected (14, 15). Moreover, tobacco smoking up-regulates angiotensin-converting enzyme 2 (ACE2), receptor, binding site of Sars-Cov2 on membrane, promoting cell-invasion (16). The initial lower prevalence of smokers among patients with COVID-19 in early publications were not confirmed and could be related to selection bias, inadequate tobacco smoking definition and other confounding factors such as social habits (7, 16).

As expected, younger age and unemployment were more prevalent among smokers, which could interfere with other findings such as occupational characteristics.

Smokers also reported a higher rate of COVID-19 screening, which could be a result of respiratory symptoms mimicking COVID-19 symptoms, thus justifying the request for testing. In our population, diabetes was more prevalent among smokers than non-smokers, and the association of these factors exacerbates CV risk. This underlines the importance of implementing strategies for tobacco cessation in smokers with comorbidities (7, 17).

Although the lockdown period provided a potential opportunity for smoking cessation, none of our participants had quit, a third of patients had increased their tobacco consumption, one patient started smoking, and two patients relapsed (7). Psychological distress induced by social isolation and fear of the disease may have created conditions for smoking increase during the lockdown (1, 4). In addition, weight variations were more common among smokers than non-smokers. Whether it could relate to the influence of lockdown on mental health or to other factors such as variations in physical activity, or any confounding factors including socio-economic status is only speculative (6).

In a web-survey conducted in US dual users, 28.3 and 24.9% decreased their smoking and vaping consumption, but more subjects, 30.3 and 29.1%, respectively, had increased consumption since the beginning of the COVID-19 outbreak, and there was a positive correlation between the two products (18). In England, an analysis of monthly cross-sectional surveys demonstrated the stability of smoking prevalence and found an increase quitting since the lockdown, but they could not exclude an increase in uptake or relapse (19). In a german survey, almost 10% of smokers quit and 50% increased their tobacco consumption. The increase was associated with COVID-19-related stress and living alone (20). To the best of our knowledge, our work is the first to specifically address smoking in CVD outpatients, who constitute a high-risk population.

In France during the first COVID-19-related lockdown, a nationwide web-based survey was conducted in 1,454 respondents aged 25–64 years, including some with CVD (21). When compared with our findings, they found a similar rate of smokers who decreased their tobacco consumption (22.6 vs. 20.9% respectively), but a higher rate of increased consumption (40.4 vs. 30.2%, respectively). A cross-sectional study in smokers from the general French population covering the same lockdown period yielded similar variations, including decreased tobacco consumption in 18.6% of and increased consumption in 26.7% (6). In this online survey, smoking increase was closely related with anxiety and overcrowded housing.

A large nationwide cross-sectional survey was conducted in USA smokers and e-cigarettes users during 2020 August; 21% of smokers had decreased their tobacco consumption in the 6 last months. Although they were aware of the amplified risk of COVID-19 related to tobacco smoking, 33% of smokers had increased their consumption; one the main reasons was stress; results were similar between only cigarettes users and dual-users. Moreover, 15% of the subjects who had quitted during the last 6 months relapsed. Conversely, 23% of vapers increased their e-cigarette consumption. However, 26% of smokers reported trying to quit, and this was associated with an increase risk perception of COVID-19 related to tobacco smoking (22). In California, an online survey did not find an increase in the number of smokers but tobacco consumption was higher among smokers likely related to a shift in time spent in smoke free places toward time spent at home (20). In an on-line survey in Pennsylvania, stress, more time to smoke and boredom were the main reasons to smoking increase (23).

A link between stress and unhealthy behaviors has been found in Australian subjects, of whom more than 50% suffered from chronic disease, mostly driven by a decrease in physical activity (almost 50%) (24).

In Netherlands, Van der Werf et al. observed some change in lifestyle behaviors among 1,004 adults who answered an online questionnaire after the first 3 months of COVID-19 pandemics, of whom 153 (15.2%) were smokers (25). A greater number of subjects declared healthier than unhealthier lifestyle behaviors (19.3 vs. 12.2%, respectively). Unhealthier lifestyle was associated to stress and was similar among smokers and non-smokers. Most of smokers did not change their tobacco consumption; however, 8.3% declared a decrease in tobacco consumption and only 3.7% an increase which is very different from findings from other surveys including our present study (18–24, 26). In Netherlands the lockdown rules were much less strict than in other countries including France, thus potentially influencing such findings.

Altogether, these data suggest that tobacco-smoking patterns evolution during lockdown were quite similar whatever CV health status. Although smoking has been associated with increased COVID-19 severity, studies have suggested that nicotine could be protective against SARS-COv2 infection (1, 8, 19). Our data suggest that smoking increase was not related to medical or media messages.

Smoking during lockdown was characterized by living alone, feeling cramped and urban environment. Both living alone and overcrowded housing have been associated with increased smoking, even if other socio-economic factors can interfere (6, 20). Living in a rural location during lockdown was associated with less tobacco use when compared with an urban area. Green spaces have been associated with better CV health, through reduced stress, and increased physical activity (27). However, socioeconomic factors may also influence these findings by selecting subjects with a psychological profile more prone to healthy lifestyle. An Irish study reported that increasing smoking was associated with increased alcohol intake and stress, but was not influenced by the type of residence (28). In a recent French survey, a rural residence was protective against increased screen time but not smoking (29).

Unfortunately, we did not evaluate the motivation of our patients to reduce their tobacco consumption or to quit. Among the patients with CCS, 13 declared an increase of symptoms of angina of whom two were smokers. One of them did not report a change in smoking behavior, the other declared a reduction of tobacco consumption; unfortunately, we did not assess if this reduction was related to worsening angina. These motivations have been studied among 659 smokers living in Hong-Kong. In this phone-call survey performed during the COVID-19 pandemic (while no stay-at-home orders were displayed), perceived susceptibility to COVID-19 and perceived severity of COVID-19 due to smoking were associated with likelihood of quit attempts; the authors suggested that the lower rate of perceived susceptibility than severity could be explained by medias misinformation (30, 31). Data addressing patients are however very scarce. Although not detailing their health conditions, Rigotti et al. conducted a survey enrolling post-hospitalized smokers wishing to quit; among these patients, 32% have increased their tobacco consumption since the beginning of the pandemic (mainly because of stress) and 31% have decreased or stopped; these latter behaviors were associated with increase in perceived risk of COVID-19 or developing severe infections (32). Interestingly, Gold et al. have evaluated motivations to reduce or quit smoking through an online survey. Among the 103 daily smokers, 88.3% declared one or more comorbidities - including cardiovascular diseases, known to be associated with severe COVID-19 patterns. The main reasons of reducing their tobacco consumption (68.9% of the subjects) were health concerns (33).

We acknowledge some limitations in our study. Our study was conducted at the beginning of the pandemic and thus the design was only exploratory and hypothesis-free, given the uniqueness and the previously unknown magnitude of the subsequent lockdown. However, given the consistency of our data, in agreement with current literature, we think our works provide contributory findings on this high health impact topic.

The present data were obtained by self-reporting, so we cannot exclude a reporting bias for the declaration of behaviors such as smoking and alcohol consumption, screentime, physical activity or weight. Some randomly-selected patients could not be included because they declined to participate in the study, could not be reached by phone or because of language barriers. However, the participation rate was high (86%) and the characteristics of the study population, consistent with contemporary data (13) suggest the representativeness of the study population.

Because of the small sample of smokers in our cohort, an extrapolation of our results to other population is only speculative. However, our findings are consistent with larger French general populations covering the same lockdown period, thus strongly suggesting the representativeness of our study population (6, 21).

As cardiovascular risk gradually increases with daily tobacco consumption even for one cigarette, we did not perform a quantitative evaluation of cigarette consumption (34).

Our study did not assess cardiovascular outcomes, which was out of our scope, thus we were not able to analyse the prognosis in subjects who increased their tobacco consumption.

In conclusion, CVD patients had a high rate of smoking during the 1st COVID-19 related lockdown; their behaviors were characterized by a triad of factors: psychological, socio-demographic and living environment. Moreover, the frequent increase in smoking (30%), mainly driven by stress, was particularly alarming in patients with diabetes, suggesting that more aggressive lifestyle management is needed. A longitudinal extension of this cross-sectional survey could provide relevant information regarding the duration of the behaviors described herein and their longer-term health consequences. If confirmed by large sample or experiment design, our findings may help to target tailored preventive strategies in this high-risk population.

## Data Availability Statement

The original contributions presented in the study are included in the article/Supplementary Material, further inquiries can be directed to the corresponding author.

## Ethics Statement

The present study complied with the Declaration of Helsinki and was approved by the Ethics Committee of the Dijon University Hospital (NCT04390126). The patients/participants provided their oral informed consent to participate in this study.

## Author Contributions

FC, MB, J-CE, ND, YC, and MZ: conceptualization. MS-J, AS, and GL: methodology. YC: funding acquisition. FC, FB, and MS-J: data acquisition. J-CE, FB, AC, and AS: analysis. MB, AC, ND, and YC: project administration. FC and MZ: writing draft. FC, MB, ND, YC, and MZ: writing, review, and editing. All authors has approved the submitted version and agrees to be personally accountable for its own contribution.

## Funding

This work was supported by the Dijon Football Côte d'Or, Dijon University Hospital, the French Federation of Cardiology, the Association de Cardiology de Bourgogne, and by grants from the Agence Régionale de Santé (ARS) de Bourgogne-Franche-Comté and from the Regional Council of Burgundy-Franche-Comté.

## Conflict of Interest

FC reports having received non-financial support and speaking fees from Amgen, MSD, Novartis and Pfizer. MB reports having Consulting or Advisory Role from Sanofi Aventis, BMS, Pfizer, Pierre Fabre, Grünenthal, Celgene. ND reports having received grants, speaking fees, consulting fees, or non-financial support from: Amgen, AstraZeneca, Bayer, BMS, Boehringer-Ingelheim, Intercept, MSD, Novo-Nordisk, Pfizer, Sanofi, Servier, UCB and Vifor. YC reports having received grants, consulting fees, honoraria and/or delivering lectures for Servier, Novartis, Boehringer, Pfizer, MSD, and Bayer. MZ received research grants from Amarin Corp. The remaining authors declare that the research was conducted in the absence of any commercial or financial relationships that could be construed as a potential conflict of interest.

## Publisher's Note

All claims expressed in this article are solely those of the authors and do not necessarily represent those of their affiliated organizations, or those of the publisher, the editors and the reviewers. Any product that may be evaluated in this article, or claim that may be made by its manufacturer, is not guaranteed or endorsed by the publisher.
