# Supplementary material for: Smoking in Patients With Chronic Cardiovascular Disease During COVID-19 Lockdown
Source: Front Cardiovasc Med. 2022 Apr 26;9:845439. doi: 10.3389/fcvm.2022.845439 (PMC9086588; doi:10.3389/fcvm.2022.845439)
Supplement: Supplementary file 1 [file Data_Sheet_1.pdf]

Supplementary Material 1: Questionnaire.

CLEO-CD Questionnaire

First name /__________________/ Last name /________________________/

Date of birth ___/______/________ Sex M ☐ F ☐

Marital status and living conditions before the lockdown

Married, conjugal life	☐	Living with a partner	☐
Divorced	☐	Living with other family members	☐
Widower	☐	Living with other people	☐
		Living alone	☐
		Living in retirement/nursing home	☐

Socioprofessional status before the lockdown

1/ Professionally active: ☐ Yes ☐ No

If yes, current job: /____/

2/ Work-time : ☐ Full-time ☐ Part-time (If Part-time, what %____)

3/ Type of employment contract/activity ☐ Fixed term ☐ permanent contract ☐ Liberal (If other, describe _______________)

4/ If not active, tick the corresponding box: ☐ Retired ☐ Unemployed

☐ Other not active, describe /____/

Last job /____/

Highest diploma

No diploma	☐
Primary school leaving certificate	☐
Junior secondary school diploma	☐
Certificate of professional competence, diploma of occupational studies	☐
High school diploma, professional certificate	☐
Undergrate degree	☐
Graduate degree: Master, PhD…	☐
Foreign diploma	☐
Other (describe)	/__________________________/

During the lockdown

(from March 17th to the interview day)

Lockdown

1) Do you comply with the regulations on lockdown rules : ☐ Yes ☐ No

2) Your current professional place (if any): (*Only one possible answer*)
☐ Not the usual work place ☐ Teleworking ☐ Usual work place

☐ Not applicable

3) Living conditions during the lockdown : (*Only one possible answer*)
Living alone ☐

Living with other people ☐ + /___/ (*number*)

Living in retirement/nursing home ☐

4) Your lockdown place :
(*Only one possible answer*)
House with garden : ☐ House without garden: ☐

Flat without terrace or garden ☐ Flat with terrace or garden ☐

Surface /___//___//___/ m²

Do you feel cramped: ☐ Yes ☐ No

Lockdown area (*Only one possible answer)*
Rural (< 2,000 inhabitants): ☐
Urban (>2,000 inhabitants): ☐

Current feeling

How do you currently feel *(Only one possible answer)***?**

Bad ☐ Not too bad ☐ Good ☐ Very good ☐

Do you feel worse than before the lockdown? :

☐ Yes ☐ No

Your distress on a scale from 0 to 10 : /___//___/
0 = No distress to 10 = Maximum imaginable distress

Your status regarding COVID-19

Did you had a COVID-19 nasal test *(rt-PCR test)* **?**

☐ Yes ☐ No

If yes, date: ___/_____/_______

Results: COVID-19 Positive ☐ COVID-19 Negative ☐ ☐ I do not know
If no, has anyone that you recently met (within the last month) had caught COVID-19?

☐ Yes ☐ No

If yes, when did you met him (her) for the last time? (*delay in days*)? /___//___/ d

Did you experience any abnormal symptoms during these last days or weeks? *(one or several responses)*

a. Fever	☐ Oui	☐ Non
b. Unusual cough	☐ Oui	☐ Non
c. Unusual breathlessness	☐ Oui	☐ Non
d. Myalgias	☐ Oui	☐ Non
e. Loss of smell and/or loss of taste	☐ Oui	☐ Non
f. Headache	☐ Oui	☐ Non
g. Palpitations	☐ Oui	☐ Non
h. Unusual chest pain	☐ Yes	☐ No

Physical activity

Since the beginning of the lockown, how much time do you spend on physical activity?
(Only one possible answer)

More ☐
Same ☐
Less ☐

If more, slightly ☐ moderately ☐ a lot ☐

Smoking and vaping (at the time of interview)

Do you currently smoke? (daily or occasional)

☐ Yes ☐ No

-If no *(Only one possible answer)*:

1. You were non smoker or ex-smoker ☐

2. You stopped since the beginning of the lockdown ☐

-If yes *(Only one possible answer)*:

1. You already smoked before the lockdown ☐
In this case, when compared with before lockdown, you currently smoke:
☐ less
☐ as much

☐ more, if yes why ?
☐ Medical counselling
☐ Media/social net/web information
☐ Other, describe ______________________________

2. You did not smoke before the lockdown and you started smoking ☐

if yes why?
☐ Medical counselling
☐ Media/social net/web information
☐ Other, describe ______________________________

3. You had previsously quit smoking and you relapsed ☐

if yes, why?
☐ Medical counselling

☐ Media/social net/web information
☐ Other, describe ______________________________

17) Do you currently vape?
(e-cigarette)

☐ Yes ☐ No

-If no *(Only one possible answer)*:

1. **You never vaped or you are ex-vaper** ☐

2. You stopped vaping since the beginning of pandemic or lockdown ☐

-If yes, vape type:

☐ with nicotine ☐ without nicotine
(Only one possible answer)

1. You already vaped before the lockdown ☐
In this case, when compared with before lockdown, you currently vape:
☐ less
☐ same

☐ more, if yes why ? ☐ Medical counselling
☐ Media/social net/web information
☐ Other, describe ______________________________

2. You did not smoke before the lockdown and you started smoking ☐

if yes why? ☐ Medical counselling
☐ Media/social net/web information
☐ Other, describe ______________________________

3. You quitted vaping and you relapsed ☐

if yes, why? ☐ Medical counseling
☐ Media/social net/web information
☐ Other, describe ______________________________

At risk factors and behaviors

Do you suffer from diabetes?

☐ Yes ☐ No ☐ I do not know

If yes, is it currently less well stabilized? ☐ Yes ☐ No ☐ I do not know

What are your last blood pressure values?

Value (mmHg): SBP: /___//___/ DBP: /___//___/

I do not know ☐

Since the lockdown, your daily alcohol intake *(including wine, beer, aperitifs, spirits etc.)***:**

☐ increased ☐ decreased ☐ did not change

Since the lockdown, has your sleep quality and/or quantity decreased?

☐ Yes ☐ No

Since the lockdown, has your screentime increased?
(whatever the screen: television, computer, electronic tablet, cell phone)

☐ Yes ☐ No

Your body weight

-Last value before the lockdown: /___//___/ Kg or ☐ I do not know

Your height /___/ , /___//___/ in Meters or ☐ I do not know

From the beginning of the lockdown, your body weight:

☐ has increased ☐ has decreased ☐ did not change ☐ I do not know

-Your current body weight : /___//___/ Kg or ☐ I do not know

24) Before the lockdown, did you experience a depressive episode that needed medical management**?**

☐ Yes ☐ No

If yes, which kind of management? *(one or several responses)*

☐ psychological/psychiatric management ☐ pharmacological
☐ Other, please describe /____/

Psychologic distress scale (K6)

(Kessler, Psychol Med 2002)

The following 6 questions address to how you feel since the beginning of the lockdown (March 17)

For each question, tick the response that best describes your feeling
(Only one possible answer per question).

Since the beginning of the lockdown (March 17), how many times did you feel…

1.…nervous?

☐ Always ☐ Most of the time ☐ Sometimes ☐ Rarely☐ Never

2.…hopeless?

☐ Always ☐ Most of the time ☐ Sometimes ☐ Rarely☐ Never

3. …restless or fidgety ?

☐ Always ☐ Most of the time ☐ Sometimes ☐ Rarely☐ Never

4 **…so depressed than nothing could cheer you up?**

☐ Always ☐ Most of the time ☐ Sometimes ☐ Rarely☐ Never

5. …that every thing was an effort?

☐ Always ☐ Most of the time ☐ Sometimes ☐ Rarely☐ Never

6. …worthless ?

☐ Always ☐ Most of the time ☐ Sometimes ☐ Rarely☐ Never
